# Toxic Effects of Rare Earth Elements on Human Health: A Review

**DOI:** 10.3390/toxics12050317

**Published:** 2024-04-26

**Authors:** Wenyu Wang, Yanfang Yang, Donglei Wang, Lihua Huang

**Affiliations:** School of Public Health, Baotou Medical College, Baotou 014030, China; 18503209703@163.com (W.W.); yangyanfang96@163.com (Y.Y.); wang19971222@126.com (D.W.)

**Keywords:** rare earth element, human health, toxicity effect, toxicity mechanism

## Abstract

Rare earth elements (REEs) are a new type of material resource which have attracted significant attention in recent years. REEs have emerged as essential metals in modern-day technology due to their unique functions. The long-term, large-scale mining and utilization of rare earths has caused serious environmental pollution and constitutes a global health issue, which has raised concerns regarding the safety of human health. However, the toxicity profile of suspended particulate matter in REEs in the environment, which interacts with the human body, remains largely unknown. Studies have shown that REEs can enter the human body through a variety of pathways, leading to a variety of organ and system dysfunctions through changes in genetics, epigenetics, and signaling pathways. Through an extensive literature search and critical analysis, we provide a comprehensive overview of the available evidence, identify knowledge gaps, and make recommendations for future research directions.

## 1. Introduction

REEs refer to a series of elements which includes lanthanides, Sc, and Y, which is made up of seventeen metallic elements in the periodic table [1]. REEs are usually divided into two groups based on their different structures and characteristics. La, Ce, Pr, Nd, Pm, Sm, and Eu are referred to as light rare earth elements (LREEs), while Gd, Tb, Dy, Ho, Er, Tm, Yb, Lu, Sc, and Y are referred to as heavy rare earth elements (HREEs) [2]. Major REE mining areas around the world include Bayan Obo in China, Mountain Pass in the United States, Mount Weld in Australia, REE deposits in eastern Canada, and ion-adsorption deposits in several Chinese southern provinces [3,4]. Over the last few decades, REEs have been synthesized and used in various industries due to their characteristics [5]. However, numerous studies have shown that the long-term, large-scale exploitation and utilization of REE minerals may lead to excessive REE content in atmospheric particulate matter [6]. Epidemiological studies have suggested that Baiyan Obo in China and Nunavik in Canada are areas with severe REE pollution in the environment [7,8]. Among them, the polluted areas of REEs are mainly located near high-polluting factories related to mining, nearby residential areas, and junctions in cities with heavy traffic [9,10,11]. People in areas contaminated with REEs can be exposed to significant amounts of REEs through their skin and inhalation. REE accumulation has been detected in human blood, urine, and hair, suggesting that long-term exposure to REEs has potential risks to human health [12,13]. Meanwhile, excessive REE levels in food can also be ingested and can lead to REE accumulation in the digestive tract [14,15,16]. In addition, REE exposure is not limited to the vicinity of mining sites. With the rapid development of the medical industry, iatrogenic exposure has also become an important route for REE exposure [17,18].

REEs can enter the human body through multiple exposure routes and accumulate in different tissues or organs, ultimately posing a threat to human health [19]. The aforementioned findings clearly show that REE exposure is a public health issue of global importance. However, there has been relatively little exploration of the toxicological effects and mechanisms of REEs’ effects on human health [20]. Therefore, the main purpose of this review is to focus on the current literature to provide an overview and discuss the hazardous effects of REE exposure on human health.

## 2. Materials and Methods

### 2.1. Search Strategy

We conducted a literature review using the PubMed and Web of Science databases to identify articles on the toxicity of rare earth exposure to health published up to December 14, 2023. The retrieval strategy was as follows:

#1: (Earth Metals, Rare) OR (Rare Earth Metals) OR (Rare Earth Metal) OR (Earth Metal, Rare) OR (Metal, Rare Earth) OR (Lanthanoid Series Elements) OR (Cerium) OR (Dysprosium) OR (Erbium) OR (Europium) OR (Gadolinium) OR (Holmium) OR (Lanthanum) OR (Lutetium) OR (Neodymium) OR (Praseodymium) OR (Promethium) OR (Samarium) OR (Terbium) OR (Thulium) OR (Ytterbium) OR (Scandium).

#2: (Health) OR (Individual Health) OR (Health, Individual) OR (Occupational Health) OR (Adolescent Health) OR (Child Health) OR (Maternal Health) OR (Infant Health) OR (Men’s Health) OR (Women’s Health) OR (Sexual Health) OR (Population Health) OR (Public Health) OR (Reproductive Health).

#3: #1 AND #2.

Additionally, the studies identified through the aforementioned search strategy were examined. 

### 2.2. Inclusion Criteria

Studies were included if they involved (1) epidemiological studies on the effects of REEs on human health, to explore the relationship between REEs and adverse outcomes such as respiratory and cardiopulmonary diseases; (2) research delving into the toxicity and underlying mechanisms of REEs utilizing in vivo and in vitro models with commonly utilized laboratory materials (e.g., mice, zebrafish, and human tissue cells); or (3) the comprehensive analysis of the mechanisms of REE-induced human body damage, including genetics, epigenetics, and abnormal changes in signaling pathways.

### 2.3. Exclusion Criteria

The studies were excluded if they were (1) not written in English or (2) if original data about in vivo and in vitro experiments were not available.

## 3. Rare Earth Exposure

Inhalation exposure is the most common exposure route of exposure to atmospheric particulates [21]. A study of airborne particulate matter in Baotou City estimated that the average daily intake of REEs through PM2.5 inhalation ranged from 5.09 × 10^−7^ to 2.25 × 10^−5^ mg kg^−1^ d^−1^ [22]. In particular, the daily intake of REEs by residents in mining areas was found to be much higher than that of residents in non-mining areas [23]. An epidemiological investigation showed that the average dose of REEs inhaled by residents of a mining area was as high as 101.03 to 430.83 μSvyear^−1^ [24]. Long-term exposure to inhaled REE particles can lead to significant REE deposition in the lungs [25,26]. REE particles can also enter the human body through hair follicles and sweat glands, causing bodily damage [27]. In long-term exposure to an environment with excessive REE content, REEs can also cross the placental barrier and cause intrauterine damage to a fetus through accumulation [28]. Recent studies have shown that the production of global waste of electrical and electronic equipment (WEEE) rich in REEs has increased significantly, further driving environmental pollution and creating threats to human health caused by REEs [29]. This is due to these new pollutants having nondegradable components and having long half-lives [30,31,32]. The oral inhalation of REE can lead to long-term deposition in humans and produce chronic toxic effects [33]. In addition, WEEE is spread through the air and in other ways, which also means exposure to rare earth elements is no longer limited to residents in mining areas [34]. Meanwhile, since intravascular gadolinium contrast agents are used as substitutes for iodine contrast agents, the potential toxicity of REEs to the human body through iatrogenic exposure cannot be ignored [18]. In conclusion, the multiple exposure pathways of REEs and their consequent health risks have attracted our attention. 

## 4. Rare Earth Toxicity

By searching for and summarizing relevant studies published in recent years, we found that exposure to REEs in the environment can harm human health [35]. Toxicological effects due to the bioaccumulation of REEs have been extensively evaluated in a large number of in vivo and in vitro models [20,36]. However, the current understanding of REEs is relatively limited, and these studies have only briefly explored the interaction between the toxic effects of certain REEs and human health. We further systematically explored and integrated toxicity studies of REE exposure that explored respiratory, cardiovascular, neurological, reproductive, and other unclassified systems. Table 1 provides a summary of REEs associated with human health hazards.

### 4.1. Respiratory System

Although atmospheric particles can be cleared by the immune mechanism of the human body, some REEs remain in the respiratory tract and produce toxic effects [55]. A number of observational studies on exposed populations have pointed out that workers who inhaled REE particles have a significantly increased incidence of airway and interstitial lung diseases, such as inflammation, granulomatous degeneration, pulmonary fibrosis, pneumoconiosis, and even cancer [37,38,56], which may be caused by the accumulation and irritation of REEs. Based on epidemiological results, numerous animal experiments simulating exposure levels of REEs have found that REEs can indeed cause severe lung damage. For example, Snow et al. showed that REE particles can be deposited in the lung through respiration, activating oxidative stress and inducing pulmonary inflammation in mice [57,58,59]. In vivo experiment, granulomatous changes appeared in the lung tissue of rats when the concentration of cerium nitrate was increased to 75 mg/kg body weight/day [60,61]. Respiratory function disruption caused by the long-term intratracheal instillation of REEs led to restricted ventilation dysfunction in mice and eventually transformed into interstitial pulmonary fibrosis [39,62]. In addition, in vitro experiments further confirmed that REEs could enter lung cells and lead to decreased cell viability and enhanced apoptotic ability through reactive oxygen species (ROS) production and DNA damage effects [63]. Notably, the adverse effects of REEs on lung cells are influenced by environmental factors, particle size, exposure dose, and duration [20,64,65]. In particular, long-term exposure to nanoscale REE particles can cause more serious damage to the lungs [40].

### 4.2. Nervous System

REEs can cross the blood–brain barrier and deposit in the brain, which underlies their neurotoxicity [66,67]. Epidemiological investigations of residents living in mining areas have shown that long-term exposure to REEs can cause neurological diseases, such as motor and sensory impairments, neurodegeneration, or neurosis [43,68]. Observational studies in special populations, such as children and pregnant women in mining areas, have shown that REEs can lead to reduced intelligence and motor ability in children, can deposit in the fetal brain, and can affect neural tube development [44,69]. These studies have shown that REEs can be deposited in the brain, impair the development of the central nervous system, and even pass through the placental barrier to generate passage or transgenerational inheritance. A series of in vivo studies of neurological disorders associated with REE exposure have been reported by several groups. REEs were found to be able to deposit in the cerebral cortex and hippocampus, causing a significant reduction in plasma neurotransmitter levels and the number of neurons in mice, leading to impaired motor ability, spatial recognition, and memory [45,70,71]. Xu et al. found in Caenorhabditis elegans that REEs can cause neurodegenerative changes by inducing damage to dopaminergic and GABAergic neurons [72]. In addition, REEs can cause depression, anxiety, and sample behavior in mice, confirming that REE exposure can cause severe neurosis [73]. In vitro studies have also shown that REEs are deposited in human neurons and exert effects on neuron cell viability, morphology, apoptosis, and mitochondrial respiratory function [74], further revealing that REE exposure is associated with nervous system damage.

### 4.3. Cardiovascular System

Studies have shown that long-term exposure to REEs can cause leukopenia, increase telomerase activity in human peripheral blood monocytes, and even lead to lymphoma and leukemia [75,76]. The results of a cross-sectional study showed that children and adolescents in mining areas had lower blood levels of trace elements and hemoglobin, resulting in an increased probability of anemia [46]. These results indicate that REEs can be deposited in the blood and affect the number and classification of cells in the blood, causing harm to human health. The toxicological effects of REEs on the cardiovascular system were evaluated in animal models. REEs can be deposited in mice, reduce the number of blood cells, and induce inflammatory cell aggregation and the release of pro-inflammatory factors, leading to hematopoietic function and vasoconstriction disorders [47,77,78]. Zhao et al. also reported pathological changes in zebrafish after REE exposure, such as pericardial edema, cardiac contraction disorders, and myocardial hypertrophy [41], suggesting that REEs have adverse effects on the structure and function of the cardiovascular system. REEs induce abnormal vascular development in zebrafish by activating the apoptotic pathway [48]. In addition, Gojova et al. found that as markers of inflammation, intercellular cell adhesion molecule-1, interleukin-8, and monocyte chemotactic protein-1 were significantly increased in human aortic endothelial cells that internalized REE particles, suggesting that REEs can induce inflammation in vascular endothelial cells [79]. REEs can activate oxidative stress, induce inflammatory responses, and damage endothelial cells, leading to atherosclerosis [80,81].

### 4.4. Reproductive System

The adverse effects of REEs on reproductive health have been a controversial topic. Studies have shown that the effects of REEs on male reproduction include impaired spermatogenesis, reduced sperm quality, and testicular tissue damage [42,82]. Animal experiments confirmed that there was a significant positive correlation between the deposition of REEs in the testes of mice after long-term exposure to 0, 20, and 40 mg/kg REEs and the degree of sperm DNA damage and exposure dose [49]. This may be related to inflammation, oxidative stress, and disruption of the blood–testis barrier. Similarly, increased REE levels in women’s serum may adversely affect the outcome of in vitro fertilization–embryo transfer and increase the risk of spontaneous abortion [83]. Numerous animal studies have found that REEs can be deposited in the placental trophoblastic layer of mice exposed to REEs, potentially leading to adverse pregnancy outcomes including placental dysfunction, fetal loss, or growth restriction [84]. In addition, there is an association between REE exposure and severe fetal and neonatal injury. REE exposure during pregnancy can lead to fetal cleft lip and palate and an increased risk of stillbirth or neonatal death [50,85]. Studies on pregnant mice exposed to REEs found that the number of primary follicles in newborn mice was significantly suppressed, suggesting that REEs may cause reproductive toxicity in the passage [86].

### 4.5. Other Systems

In addition to the above systems, the potential toxicity of REEs to the human body involves other systems. After long-term exposure to REEs, REE deposition can be detected in bone tissue, which reduces bone density and interferes with bone metabolism, leading to osteoporosis and bone and joint injury [51,52,53]. This is because the influence of REEs can directly replace Ca^2+^ calcium phosphorus metabolism or can indirectly regulate the osteoclast combination of Ca^2+^ receptor-induced osteoporosis [54,87]. Large deposits of REEs were also detected in patients with liver injuries in a mining area, and there was a U-shaped relationship between serum REE levels and oral cancer risk, indicating that large doses of REE exposure can cause gastrointestinal injury [88,89]. Hao et al. pointed out that REEs can increase the burden of renal clearance of metabolites and cause damage to the urinary system [90]. REEs can also induce increased thyrotropin secretion, leading to histopathological changes and thyroid dysfunction [91]. In vivo experiments further confirmed that luteinizing hormone, follicle-stimulating hormone, and prolactin were significantly decreased in mice after the oral administration of REEs [92], suggesting that REEs have endocrine-disrupting effects. In addition, Martin-Aguilar et al. found a strong association between an increased number of brain MRI gadolinium enhancement lesions and multiple sclerosis recurrence, suggesting that REE exposure may lead to immune system impairment [93]. Taken together, these studies highlight the potential toxicity of REEs in various systems with adverse consequences on human health, and they contribute to the further exploration of the role of REEs in toxicology to minimize the corresponding health risks.

## 5. Toxicity Mechanisms

Although the mechanism of REE toxicity has been reported in several studies, research in this field still needs to be improved [94,95]. Many studies have indicated multiple regulatory effects in addition to oxidative damage and apoptosis. Therefore, this review mainly explores the mechanism of REE toxicity from the following aspects: genetics, epigenetics, and alterations in signaling pathways. 

### 5.1. Genetics

Firstly, DNA damage in the form of gene mutations, chromosome damage, or number change is considered to be the basic change in genetic damage, which can lead to apoptosis or necrosis. Epidemiological studies on exposed cohorts have shown that REE exposure can lead to an increase in urinary 8-OHdG, suggesting that DNA oxidative stress damage is a potential mechanism of health hazards caused by REEs [75]. A large number of in vitro and in vivo experiments have confirmed that REE exposure can directly or indirectly activate oxidative stress, induce the cleavage of DNA repair protein Poly ADP-ribose polymerase (PARP), prevent chromosome agglutination, and lead to DNA damage [96,97,98,99]. In addition, cell experiments with internalized REE particles showed an increase in DNA double-strand break marker proteins, γ-H_2_AX, and a decrease in DNA repair proteins such as p-53 and PARP, confirming that REEs can induce genetic changes and cause DNA damage [100,101]. 

### 5.2. Epigenetics

Secondly, with the intensive study of epigenetics in disease development, research into non-coding RNA (ncRNA) as a molecular target has become a hot topic. High-throughput sequencing results showed that REE-induced damage was related to abnormal changes in ncRNA expression profiles [102]. For example, in a human bronchial epithelial cell model exposed to nanoparticles of neodymium oxide (NPs-Nd_2_O_3_), 1915 circRNAs (1025 up-regulated and 890 down-regulated) were abnormally expressed, inducing tissue dysfunction through sponge miRNAs [103]. The abnormal expression of lncRNAs can activate NF-κ B and induce inflammation [104]. Moreover, Let-7a miRNA and miR-34a have also been confirmed to be abnormally increased in REE-exposed cervical cancer cells [105]. In addition to this, methylation is also crucial in epigenetic modification. The results of a recent study have shown that DNA methylation levels are reduced in human fibroblast cell lines exposed to REEs, which abnormally affect cell morphology and viability [106]. REE exposure can also enhance the methylation modification of histone H3, increase the binding of the MLL1 complex in the NRF2 promoter region, and induce genotoxicity in cells [107]. 

### 5.3. Altered Signaling Pathways

In addition to the above mechanisms, classical pathways including inflammatory response, immune response, and endocrine signaling are significantly affected by REE exposure, such as abnormal changes in the Nrf2, MAPK, and Toll-like receptor (TLR) signaling pathways [108,109,110]. A cross-sectional study of e-waste site residents showed that REE exposure led to increased biomarkers of oxidative stress, suggesting that REE exposure caused endocrine diseases through increased oxidative stress, leading to hormonal changes in the hypothalamic–pituitary–thyroid axis (HPT) [91]. Similarly, REEs deposited in animals can increase intracellular ROS levels and trigger an increase in Nrf2 gene expression, which further activates the Nrf2 endogenous antioxidant pathway and induces vascular injury in mice [111]. In addition, in vitro and in vivo experiments have confirmed that REE particles can directly or indirectly activate the NF-κ B signaling pathway, promote the synthesis and release of inflammatory chemokines, enhance immune cytotoxicity, and induce inflammation [108,112,113]. Table 2 briefly summarizes the mechanisms of REE toxicity based on the results of current in vitro and in vivo studies.

## 6. Conclusions

**Figure 1:**
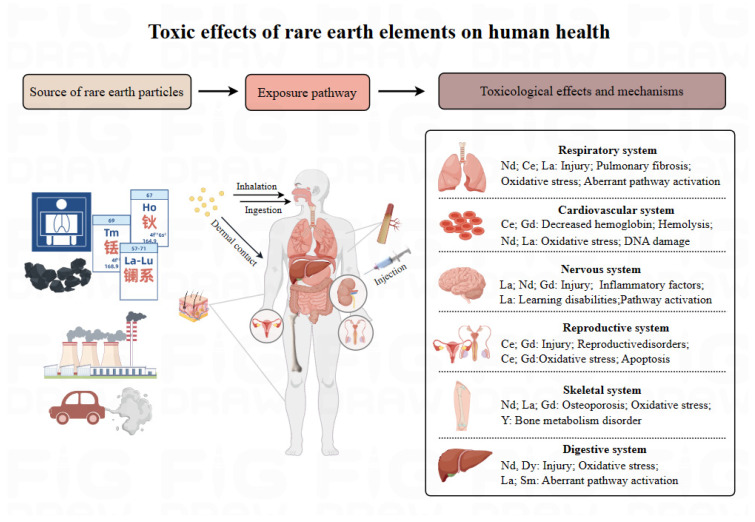
Toxic effects of rare earth elements on human health.

Due to the widespread distribution and persistence of REEs in the environment, there is an urgent need to fully understand the harmful effects and mechanisms of REE particles on human health. By comprehensively summarizing current knowledge, we found that the human body can be exposed to REEs through various pathways such as inhalation, ingestion, dermal contact, and iatrogenic exposure, and this causes deposition, which in turn destroys the structure and function of various organs of the human body and induces multi-system diseases (e.g., respiratory, nervous, cardiovascular, reproductive, and immune systems). Notably, the adverse effects of REEs on various tissues and organs are also affected by environmental factors, particle size, and exposure dose and duration. Numerous in vitro and in vivo studies have shown that REEs exert these adverse effects mainly by affecting genetics and epigenetics, altering the activation of signaling pathways (Figure 1). Although epigenetics is a promising molecular target for early diagnosis and prevention, the specific mechanisms by which REEs damage organisms are not fully understood. Through the evidence presented in this review, the correlation between exposure risk and potential health hazards of REEs was identified, which could contribute to their future development. However, the current information on the toxicological assessment of REEs is still insufficient, and there are still some challenges in finding the critical standard for human health hazards caused by REE exposure.

## 7. Challenges and Perspectives

Although REEs have become a hot spot in toxicology research in recent years, limited by the synergistic toxic effects of various REEs in the actual environment, imperfect detection indicators, and dynamic metabolic differences in different individuals, the hazards of REEs to human health are still largely unknown. Further exploration of the interaction between them is helpful to emphasize the causal relationship between toxicant exposure and pathological state, explore the detection standard and safety limit of REE exposure, and develop new molecular markers for organ damage caused by REEs. Several important issues associated with this challenge need to be addressed in this review: (1) Current studies on the interaction between REEs and health hazards are mostly limited to cell and animal models. In order to further verify the toxic effect of REE exposure on the human body, long-term epidemiological cohort studies will become the next direction of close research. (2) The safety threshold of REE exposure should be established, especially the criteria for rare earth pneumoconiosis. Moreover, REE exposure doses in daily security standards are crucial. (3) In most epidemiological studies on REE exposure, the population is made up of few subjects, meaning it is difficult to tease out the toxic effects of individual REEs in complex mixtures in human biomonitoring studies. Therefore, the time–dose–response relationship between REEs and human health hazards still needs to be further explored. Increasing the understanding of REE exposure will further elucidate the toxic effects and mechanisms of REEs and its compounds and promote the development of future toxicological-related research fields. Ultimately, this will contribute to the development of diagnostic and therapeutic measures for REE-related diseases and provide regulatory guidance for hazard assessment and exposure thresholds for REEs.

## Figures and Tables

**Table 1:** Summary of REE-associated toxicological studies.

	Element	Section Studied	Toxicity Outcome	Reference
Respiratory system	Y	Endotracheal	Dyspnea and pulmonary edema, pleural effusions	[21]
	Ce	Environmental exposure, skin contact	Extrapulmonary translocation, interstitial lung disease, pulmonary fibrosis, pneumoconiosis, cytotoxicity	[37]
	La	Environmental exposure	Phosphate deposition, pulmonary fibrosis	[38]
	Ce	Occupational exposure, environmental exposure	Cytotoxicity, genotoxicity, lung cancer, inflammation, granulomas, mobilization	[39]
	Dy	Endotracheal instillation	Lung injury, oxidative stress, inflammatory response	[40]
	Sm	Endotracheal	Lung injury, inflammatory response, pulmonary fibrosis	[41]
	Th	Environmental exposure, skin contact	Dyspnea, pneumoconiosis, lung cancer	[42]
Nervous system	Gd	Iatrogenic exposure	Deposits in the brain, brain damage	[43]
	Nd	Environmental exposure, food chain	Fetal neural tube defects	[44]
	La	Environmental exposure, skin contact, food chain	Learning and memory impairment, decreased spatial discrimination, cytotoxicity, memory disorders	[45]
Cardiovascular system	Ce	Environmental exposure,	The hemoglobin level is reduced, anemia	[46]
	Gd	Endotracheal instillation	Cytotoxicity, hematopoietic destruction	[47]
	La	Occupational exposure	Deposition in blood vessels	[41]
	Nd	Environmental exposure, skin contact, food chain	DNA damage, cytotoxicity, abnormal cardiovascular and cerebrovascular development	[48]
Reproductive system	Ce	Environmental exposure, oral administration	Oxidative stress, placental dysfunction, fetal abortion, growth restriction	[49]
	Gd	Iatrogenic exposure	Inflammatory or invasive skin diseases, stillbirth, neonatal death	[50]
Skeleton	Gd	Iatrogenic exposure	Bone deposits, osteoporosis	[51]
	Y	Iatrogenic exposure	Bone deposits	[52]
	Nd	Occupational exposure, environmental exposure	Disorders of bone metabolism, decreased bone mineral density	[53]
	La	Environmental exposure, food chain	Abnormal metabolism of calcium and phosphorus, decreased bone mineral density	[54]

Note: REE, rare earth element; Y, yttrium; Ce, cerium; Dy, dysprosium; La, lanthanum; Nd, neodymium; Gd, gadolinium; Sm, samarium; Th, thorium; Yb, ytterbium.

**Table 2:** Summary of related toxicological mechanisms of rare earth elements.

	Type	Sample	REE Exposure	Toxicity	Reference
Genetic	In vivo	C57-ras	12.5, 25, and 50 mg/kg lanthanum nitrate for 180 d	Rare earth deposition causes direct damage	[66]
	In vitro	SH-SY5Y	10, 25, 50, and 100 µg/mL Gd_2_O_3_ for 24 and 48 h	Apoptosis is regulated by bcl-2/bax protein expression	[98]
	In vivo	Rat	1 mg/kg CeO_2_ for 6 d	Oxidative stress, inflammation, DNA damage	[111]
Epigenetic	In vitro	16HBE	0, 5, 10, 20, 40, and 80 μg/mL Nd_2_O_3_ for 6, 12, 24, 48, and 72 h	circ_009773 regulates DNA damage	[103]
	In vitro	16HBE	10 μg/mL NPs-Nd_2_O_3_ for 48 h	Promotes NF-κ B activation and promotes cellular inflammation by negatively regulating adiponectin receptor 1 expression	[104]
	In vitro	Human fibroblast cell	0.05 to 1.6 mg/mL of Tb-MOF for 48 h	Altered gene methylation, induced genetic damage	[106]
Signaling pathways	In vivo	Rat	0 and 1 mg/kg CeO_2_ nanoparticles for 6 d	Activation of oxidative stress and Nrf2 signaling pathways	[111]
	In vivo	Rat	0, 1.56, 3.125, 6.25, 12.5, 25, 50, and 100 µg/mL Nd_2_O_3_ for 24 h	Activating the NF-κ B and caspase-3 signaling pathways, promoting the synthesis and release of inflammatory chemokine	[112]
	In vivo	C57BL/6J mice	Long-term exposure to cerium nanoparticles	Activation of the NF-κ B signaling pathway can increase the cytotoxic activity of immune cells	[113]

Note: REE, rare earth element; La, lanthanum; Gd, gadolinium; Ce, cerium; Nd, neodymium; Tb, terbium.

## Data Availability

Not applicable.
